# Ablation of the *miR-465* Cluster Causes a Skewed Sex Ratio in Mice

**DOI:** 10.3389/fendo.2022.893854

**Published:** 2022-05-23

**Authors:** Zhuqing Wang, Nan Meng, Yue Wang, Tong Zhou, Musheng Li, Shawn Wang, Sheng Chen, Huili Zheng, Shuangbo Kong, Haibin Wang, Wei Yan

**Affiliations:** ^1^ Department of Physiology and Cell Biology, University of Nevada, Reno School of Medicine, Reno, NV, United States; ^2^ The Lundquist Institute for Biomedical Innovation at Harbor-UCLA Medical Center, Torrance, CA, United States; ^3^ Reproductive Medical Center, The First Affiliated Hospital of Xiamen University, Xiamen, China; ^4^ Fujian Provincial Key Laboratory of Reproductive Health Research, School of Medicine Xiamen University, Xiamen, China; ^5^ Department of Medicine, David Geffen School of Medicine at University of California, Los Angeles, Los Angeles, CA, United States

**Keywords:** *miR-465*, microRNA, sexual dimorphism, sex ratio, extraembryonic tissues, placenta, CRISPR-Cas9

## Abstract

The X-linked *miR-465* cluster is highly expressed in the testis, sperm, newborn ovary, and blastocysts as well as in 8-16 cell embryos. However, the physiological role of the *miR-465* cluster is still largely unknown. This study aims to dissect the role of the *miR-465* cluster in murine development. Despite abundant expression in the testis, ablation of the *miR-465* miRNA cluster using CRISPR-Cas9 did not cause infertility. Instead, a skewed sex ratio biased toward males (60% males) was observed among *miR-465* KO mice. Further analyses revealed that the female conceptuses selectively degenerated as early as embryonic day 8.5 (E8.5). Small RNA deep sequencing, qPCR, and *in situ* hybridization analyses revealed that the miRNAs encoded by the *miR-465* cluster were mainly localized to the extraembryonic tissue/developing placenta. RNA-seq analyses identified altered mRNA transcriptome characterized by the dysregulation of numerous critical placental genes, e.g., *Alkbh1*, in the KO conceptuses at E7.5. Taken together, this study showed that the *miR-465* cluster is required for normal female placental development, and ablation of the *miR-465* cluster leads to a skewed sex ratio with more males (~60%) due to selective degeneration and resorption of the female conceptuses.

## Introduction

Sexual dimorphism refers to different characteristics beyond the sex organs between the two sexes within the same species, e.g., appearance, structure, behavior, etc. (1). Data from a recent study of 14,250 wild-type (WT) and 40,192 mutant mice suggest that 9.9% of qualitative and 56.6% of quantitative traits display sexual dimorphism (2). Sexual dimorphism commences as early as embryonic development, e.g., X chromosome inactivation in the female embryo. Sexual dimorphism is also reflected by differential gene expression profiles in placental, fetal, and adult tissues (3–5). To date, a role of miRNAs in sexual dimorphism has not been reported although miRNAs are well known to be critical for early development (6–9). miRNAs are ~22 nucleotide small non-coding RNAs that regulate gene expression at post-transcriptional levels (10). Inactivation of either DICER or DROSHA, the two enzymes required for miRNA biogenesis, leads to early embryonic lethality in mice, indicating an essential role of miRNAs in early development (6–9, 11–13). Our previous studies have shown that the X-linked *miR-465* cluster, which encodes 6 pre-miRNAs and 12 mature miRNAs, belongs to a large X-linked *miR-506* family (14). Their high abundance in the testis, sperm, newborn ovary, blastocysts, and 8-16-cell embryos (14–17) suggests a potential role in gametogenesis and early embryonic development in mice. However, their physiological role has not been investigated *in vivo*. Here, we report that the *miR-465* cluster miRNAs are also abundantly expressed in the developing placenta, and ablation of the *miR-465* cluster does not affect fertility but causes a skewed sex ratio favoring males due to selective degradation of the female placenta during early embryonic development.

## Materials and Methods

### Generation of *miR-465* KO Mice

The animal use protocol was approved by the Institutional Animal Care and Use Committee (IACUC) of the University of Nevada, Reno (Protocol# 00494). Generation of global *miR-465* KO mice and mouse genotyping were performed as described (14, 18, 19). gRNA and genotyping primers are listed in **Table S1**.

### DNA and RNA Isolation, Library Construction, and qPCR Analyses

DNA and RNA were extracted from WT and KO embryos using the mirVana™ miRNA Isolation Kit as previously described (19). The sexes of the conceptuses were determined based on PCR amplification of *DYzEms3* (a Y chromosome-specific repetitive sequence) and *Rn18s* (a housekeeping transcript as the internal control). Males display two bands (*DYzEms3* and *Rn18s*), while females show only one band (*Rn18s*). Large RNA libraries were constructed using KAPA Stranded RNA-Seq Kits with RiboErase (Cat. # 07962282001, Roche) according to the manufacturer’s instructions. Small RNA libraries were constructed using NEBNext^®^ Small RNA Library Prep Set for Illumina^®^ (Cat. # E7330L, NEB) according to the manufacturer’s instructions. miRNA qPCR was performed as described (14). All oligos for sex determination and qPCR are listed in **Table S1**.

### *In Situ* Hybridization

Cryosections (10 μm) were adhered to poly-L-lysine-coated slides and fixed in 4% paraformaldehyde (Cat. # P6148, Sigma-Aldrich) solution in PBS for 1 h at room temperature. The sections were then washed 3 times in PBS for 5 min each, acetylated for 10 minutes (0.25% acetic anhydride), washed 2 times in PBS for 5 min each, and hybridized with DIG-labeled probes overnight at 50°C. Hybridization buffer contained 1X salts (200 mM NaCl, 13 mM Tris, 5 mM sodium phosphate monobasic, 5mM sodium phosphate dibasic, 5 mM EDTA), 50% formamide, 10% (w/v) dextran sulfate, 1 mg/ml yeast tRNA (Cat. # 10109509001, Roche), 1×Denhardt’s [1% (w/v) bovine serum albumin, 1% (w/v) Ficoll, 1% (w/v) polyvinylpyrrolidone], and RNA probe (final concentration: 1 μM). Post-hybridization washes were followed by an RNase treatment (20 μg/ml RNase A). After blocking in 20% heat-inactivated sheep serum (Cat. # ZLI-9021, Beijing Zhongshan Jinqiao Biotechnology Company) and 2% blocking reagent (Cat. # 12039672910, Roche) for 1 h, sections were incubated overnight in blocking solution containing anti-DIG antibody (1:2500 dilution; Cat. # 11093274910, Roche) at room temperature. After washing, the color was developed using NBT/BCIP according to the manufacturer’s instructions (NBT: Cat. # N1332, Gentihold; BCIP: Cat. # B1360, Gentihold). Sections were counterstained in Nuclear Fast Red (Cat. # G1321, Solarbio), dehydrated in gradient alcohol, cleared in xylene, and mounted in neutral resins. All oligos used for RNA ISH were listed in **Table S1**.

### RNA-Seq Data Analysis

The Sailfish (20) and SPORTS1.0 (21) pipelines were used to quantify the large RNA expression and small RNA expression, respectively. Transcript per million reads (TPM) was used as the unit of gene expression level. Groupwise differential expression was estimated by the likelihood ratio test and the RNAs with a false discovery rate < 5% were deemed differentially expressed.

### Luciferase Assay

Luciferase assays were performed as described (22). *cel-mir-67* was used as a negative control. *Renilla* luciferase signals were normalized to *Firefly* luciferase signals to correct the transfection efficiency. All oligos for constructing 3’UTR luciferase vectors are listed in **Table S4**.

### Statistical Analyses

Data are presented as mean ± SEM, and statistical differences between datasets were assessed by two samples t-test unless stated otherwise. p < 0.05, 0.01, 0.001, and 0.0001 are considered statistically significant and indicated with *, **, ***, and ****, respectively.

## Results

### Ablation of the *miR-465* Cluster Leads to a Male-Biased Sex Ratio

**Figure 1:**
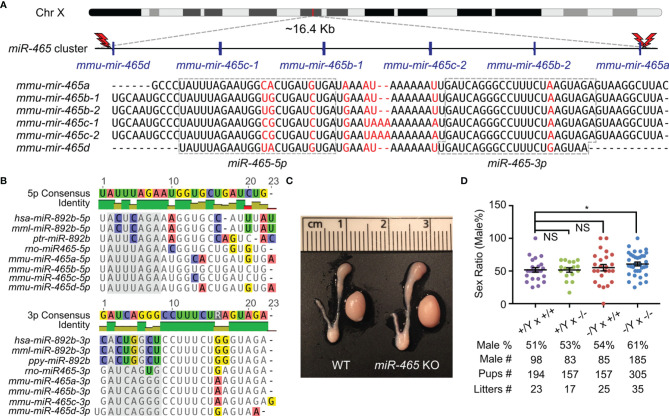
Generation of *miR-465* KO mice. **(A)** The genomic location and sequences of the *miR-465* cluster on the X chromosome of mice. The red lightning bolts represent the gRNAs used, and their right and left orientations indicate the reverse and forward strands targeted by the gRNAs, respectively. **(B)** The orthologs of the *miR-465* cluster in primates and rodents. Bases highlighted in the grey background are the potential seed regions. **(C)** A representative image of the testis and epididymis of WT and *miR-465* cluster KO mice. One unit on the ruler is 1 mm. **(D)** The sex ratios among pups from different breeding schemes. *, p<0.05; NS, statistically not significant.

The *miR-465* cluster consists of 6 miRNA genes encompassing a ~16.4 kb region on the X chromosome in mice (**Figure 1A**). Although 6 pre-miRNAs and 12 mature miRNAs are produced in mice, only 6 mature miRNAs can be distinguished based on their sequences, including *miR-465a-5p*, *miR-465b-5p*, *miR-465c-5p*, *miR-465d-5p*, *miR-465a/b/c-3p* and *miR-465d-3p* (**Figure 1A**). The *miR-465* cluster has orthologs in humans, monkeys, and chimpanzees, which have been annotated as *miR-892b* in the miRBase and contain some U-to-C or A-to-G substitutions (**Figure 1B**). Like the *miR-465* cluster, *miR-892b* is also flanked by *Slitrk2* and *Fmr1* on the X chromosome (14). To define their physiological roles, we deleted the entire *miR-465* cluster in the mouse genome using CRISPR-Cas9 (**Figures 1A**; **S1A**), as previously described (14, 18, 19). PCR genotyping and Sanger sequencing confirmed that the genomic loci of these miRNAs were successfully deleted (**Figures S1B, C**). The *miR-465* KO mice were fertile with normal testis size (**Figure 1C**). Both the litter size (8.4 ± 0.85, n=35) and litter interval (25.4 ± 0.86, n=34) of the KO mice were comparable to those of WT controls (Litter size: 8.6 ± 1.59, n=23; litter interval: 26.6 ± 1.42, n=22) (**Figure 1D**), suggesting that these miRNAs are dispensable for both spermatogenesis and folliculogenesis. Interestingly, unlike the equal distribution of the two sexes (~50%) among pups from the WT breeding pairs (*+/Y* × *+/+*), the sex ratio is significantly skewed toward the male (61%, p<0.05) among the *miR-465* KO pups derived from the homozygous breeding pairs (*-/Y* × *-/-*) (**Figure 1D**). Of interest, ~60% appears to be the most common skewed sex ratio observed in previous reports (**Table S2**) (23–28).

### The Skewed Sex Ratio Occurred During Early Embryonic Development

**Figure 2:**
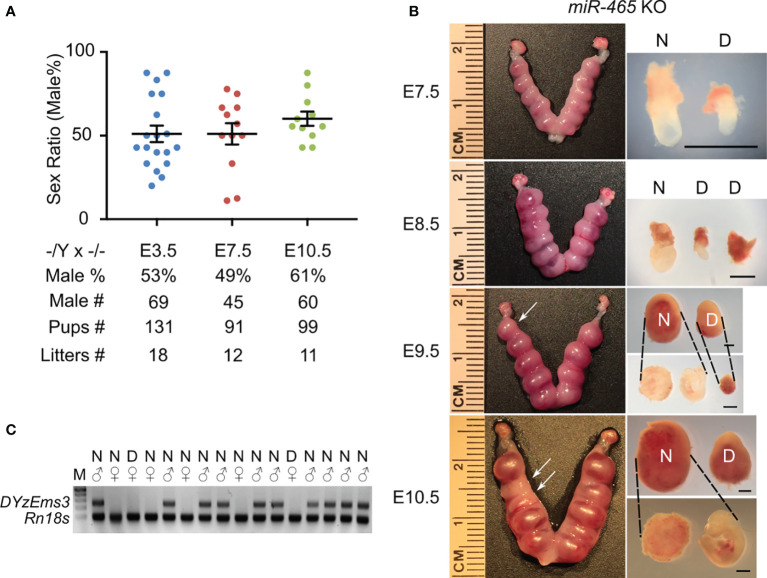
The phenotype of *miR-465* KO mice. **(A)** The sex ratios of pups from homozygous inbreeding (-/Y × -/-) at E3.5, E7.5, and E10.5. **(B)** Representative images of the KO uteri and conceptuses collected between E7.5 and E10.5. Arrows point to the degenerating/degenerated conceptuses **(D)** among the normal-looking (N) ones. Scale bars = 1mm. **(C)** A representative gel image of genotyping results. *DYzEms3*, a Y-linked genomic fragment, was amplified to identify male conceptuses, and *Rn18s*, which encodes 18s ribosomal RNA, was used as a loading control in the PCR-based genotyping analyses.

The skewed sex ratio could result from either a distorted X/Y sperm ratio or a loss of female embryos/fetuses during development. If the sex ratio is already skewed in X/Y sperm, the bias should be observed among pups from the breeding pairs of KO males (*-/Y*) and WT females (*+/+*), but not in those from the breeding pairs of WT males (*+/Y*) and homozygous KO females (*-/-*). However, the sex ratio among the pups from the *-/Y* × *+/+* breeding pairs was slightly, but not significantly, skewed toward males (54%) (**Figure 1D**), suggesting that the significantly skewed sex ratio likely occurs during development. To identify when the skewed sex ratio occurs, we collected early embryos at E3.5, E7.5, and E10.5 in the homozygous breeding pairs (*-/Y* × *-/-*). Males accounted for ~50% among all of the KO embryos at E3.5 and E7.5, whereas the ratio of the males increased to ~61% at E10.5 (**Figure 2A**), suggesting that some female embryos are lost between E7.5 and E10.5. Indeed, we observed that on average 1-2 conceptuses per uterus were either being resorbed or had already been resorbed between E8.5 and E10.5. More intriguingly, 6 out of 7 conceptuses that appeared to degenerate were all female KOs (**Figures 2B, C**). Together, these data suggest that inactivation of the *miR-465* cluster leads to selective degeneration and absorption of female conceptuses between E7.5 and E10.5.

### The *miR-465* miRNAs Are Abundantly Expressed in the Extraembryonic Tissues at E7.5

**Figure 3:**
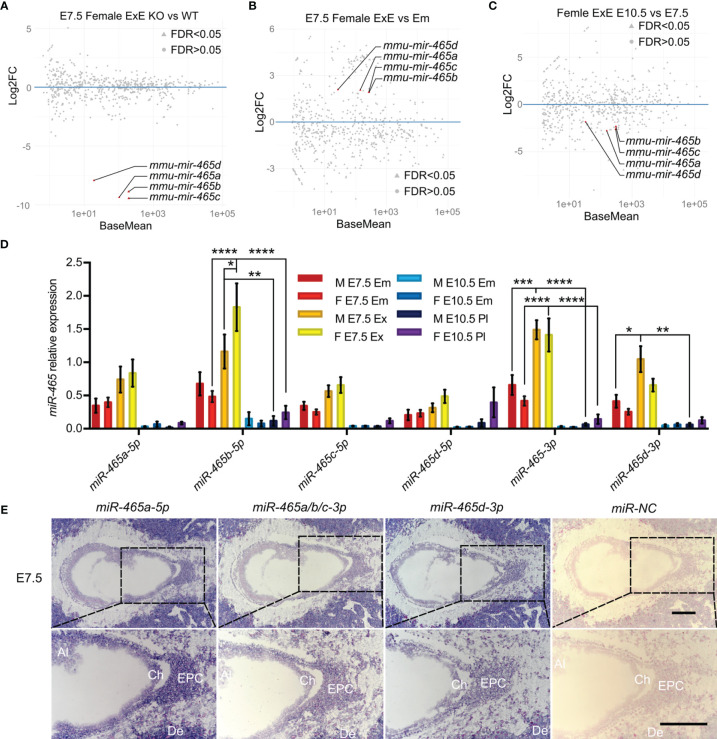
Expression profiles of the *miR-465* cluster. **(A)** Differentially expressed miRNAs between WT and KO female extraembryonic tissues at E7.5. **(B)** Differentially expressed miRNAs between WT female extraembryonic tissues and embryos at E7.5. **(C)** Differentially expressed miRNAs between WT female extraembryonic tissues/placentas at E7.5 and E10.5. Data points representing the *miR-465* cluster miRNAs are marked in red. sRNA-seq analyses were conducted in biological triplicates (n=3). **(D)** TaqMan qPCR analyses of expression levels of the *miR-465* cluster miRNAs in extraembryonic tissues/placenta and embryos at E7.5 and E10.5. M, male; F, female; Em, embryo; ExE, extraembryonic tissue; Pl, placenta. *, p<0.05; **, p<0.01; *** p<0.001, **** p<0.0001. **(E)** Representative miRNA-ISH results showing localization of the *miR-465* cluster miRNAs in female conceptuses at E7.5. Ch, chorion; EPC, ectoplacental core; Al, allantois; De, decidua. Scale bars = 200 µm.

Although the loss of the *miR-465* cluster leads to female-biased lethality, it remains unknown whether the primary defects lie in the embryos or the extraembryonic/placental tissue. To address this question, we collected both WT and KO embryos and extraembryonic/placental tissues from both sexes at E7.5 and E10.5 and performed small RNA sequencing (sRNA-seq) (**Figures 3A–C**; **S2**). sRNA-seq data confirmed that the *miR-465* cluster miRNAs were indeed absent in the KO embryos and extraembryonic/placental tissues (**Figures 3A**; **S2A–C**). While no significant sex differences in miRNA levels were observed in WT embryos and extraembryonic tissues at E7.5 (**Figures S2D, E**), the *miR-465* cluster miRNAs were predominantly expressed in extraembryonic tissues, as compared to embryos of both sexes at E7.5 (**Figures 3B**; **S2F**), and these miRNAs were significantly downregulated from E7.5 to E10.5 (**Figures 3C**; **S2G–I**). Indeed, the TaqMan real-time PCR analyses further confirmed the sRNA-seq results (**Figure 3D**). We next further performed miRNA *in situ* hybridization (ISH) assays (**Figure 3E**) to corroborate the cellular localization of the *miR-465* cluster. Consistent with the sRNA-seq and qPCR data, miRNA ISH results showed that the *miR-465* cluster miRNAs were predominantly expressed in extraembryonic tissues, especially in the ectoplacental core and chorion (**Figure 3E**). Although the *miR-465* cluster miRNAs were also detected in maternal decidua (**Figure 3E**), potential decidual defects are highly unlikely based on our breeding data showing normal sex ratio among offspring of the *+/Y* × *-/-* breeding pairs (**Figure 1D**). Given the predominant expression of the *miR-465* cluster in the extraembryonic tissues, it is highly likely that the loss of some female embryos was secondary to placental defects.

### Ablation of the *miR-465* Cluster Leads to Dysregulated mRNAs in the Female, but Not the Male, Extraembryonic Tissues

**Figure 4:**
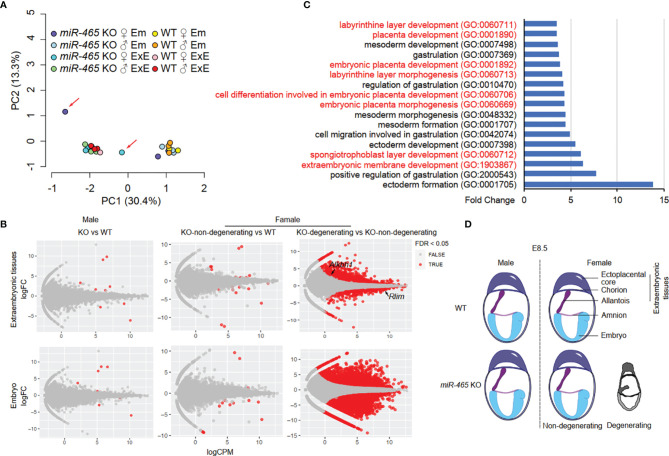
RNA-seq analyses of *miR-465* KO and WT conceptuses. **(A)** Principal component (PC) analyses of RNA-seq data from embryonic (Em) and extraembryonic (ExE) tissues of *miR-465* KO and WT mice. The red arrows indicate the degenerating embryo and extraembryonic tissues from a *miR-465* KO female conceptus. **(B)** Differentially expressed genes (DEGs) identified in the following three comparisons: between the *miR-465* KO and WT males (left), between the *miR-465* KO non-degenerating and WT females (middle), and between *miR-465* KO degenerating and non-degenerating females (right). **(C)** GO term enrichment analyses of DEGs between the degenerating and non-degenerating *miR-465* KO female extraembryonic tissues. GO terms related to extraembryonic development are highlighted in red. **(D)** Schematics showing a critical role of the *miR-465* cluster in supporting the full developmental potential of the female placenta and embryos.

To identify the targets of the *miR-465* cluster miRNAs, we then conducted RNA-seq assays on WT and KO embryos and extraembryonic tissues of both sexes at E7.5. We chose E7.5 because, at this point, despite no obvious degeneration and resorption, the transcriptomic alterations should have accumulated in the implicated female KO conceptuses (**Figure 2B**). Principal component analyses (PCA) identified two major clusters, each containing either embryos or extraembryonic tissues of both WT and most of the KO of both sexes except for two outliers (**Figure 4A**). The two outliers turned out to be one female KO embryo and its extraembryonic tissue, suggesting that this conceptus most likely represents a “to-be-degenerating” KO female. While WT and non-degenerating KO embryos and extraembryonic tissues of both sexes displayed similar mRNA transcriptomes (**Figure 4B**; **Table S3**), numerous differentially expressed genes (DEGs) were identified between the extraembryonic tissues from the “to-be-degenerating” KO female and those from non-degenerating KO females (**Figure 4B**; **Table S3**). Gene ontology (GO) term analyses identified that the DEGs were primarily involved in extraembryonic/placental development (**Figure 4C**). Among the dysregulated genes responsible for placental development, 8 out of 44 were either imprinted genes or sex-biased genes (**Table S4**). Luciferase assays further confirmed that some of the dysregulated genes were indeed the targets of the *miR-465* cluster miRNAs (**Figure S3**). Given the similar expression levels of the *miR-465* cluster miRNAs in the extraembryonic tissues of both sexes (**Figures S2D, E**), it is likely that the sexually dimorphic role of the *miR-465* cluster is achieved through miRNA-mediated post-transcriptional regulation of the sex-biased target genes. For example, *Alkbh1*, a target of *miR-465* (**Figure S3**), is a tRNA demethylation enzyme (29) highly expressed in chorion and the ectoplacental cone at E8.5 (30); its ablation also induces female-biased lethality (27).

## Discussion

The X chromosome is known to be enriched in protein-coding genes critical for reproduction and fertility (31, 32). Our earlier work has also shown that several large miRNA clusters, including the *miR-465* cluster, are either exclusively or preferentially expressed in the testis, suggesting a role in controlling spermatogenesis and male fertility (14, 33). Although ablation of the *miR-506* cluster compromises the male fertility (14), inactivation of the *miR-465* miRNA cluster does not affect either gametogenesis or fertility. Surprisingly, a lack of the *miR-465* miRNAs leads to a skewed sex ratio biased toward males due to selective degeneration of the female conceptuses between E7.5 and E10.5. Given its predominant expression in the extraembryonic tissue, the selective degeneration and absorption of female conceptuses in the absence of *miR-465* miRNAs likely reflect the compromised development of the extraembryonic/placental tissue rather than the embryos/fetuses. Therefore, the *miR-465* miRNAs appear to be required for proper development of the female, rather than the male, extraembryonic/placental tissue, supporting a role in sexual dimorphism in placental development. While sexual dimorphism is believed to mainly result from the differential gene expression between the male and female embryos (5), our study provides evidence that the placental development also displays sexual dimorphism, which can lead to a screwed sex ratio in offspring.

The 60% sex ratio seems subtle, but it is quite common in all the previous studies involving biased sex ratios (**Table S2**) (23–28). The X-linked *miR-465* cluster belongs to the SpermiRs/*miR-506* family (14, 34), and these X-linked miRNAs have no homologs on the Y chromosome. Member miRNAs of the *miR-506* family share numerous targets despite their different seed sequences (14, 34). One previous study has shown that *miR-465a-5p* is upregulated when the *miR-741* is inactivated in the cultured mouse spermatogonial stem cells (SSCs) (34), suggesting genetic compensation between these two miRNAs during spermatogenesis. Indeed, a similar phenomenon was observed in the *miR-465* KO testes. Other *miR-506* family members, including *miR-201*, *miR-463*, *miR-471*, *miR-741*, *miR-871*, *miR-883a*, and *miR-883b*, were upregulated in the *miR-465* KO testes when compared to the WT testes (**Figure S4A**; **Table S5A**). Comparisons between WT male and female extraembryonic tissues at E7.5 yielded no differentially expressed miRNAs. However, comparisons between the KO counterparts, the *miR-10a*, *miR-10b*, and *miR-196b* were upregulated in the KO male extraembryonic tissues (**Figure S4B**; **Table S5B**). Although *miR-10a*, *miR-10b*, and *miR-196b* do not belong to the *miR-506* family, they share a large number of target genes with the *miR-465* cluster (**Figure S4C**), indicating that these miRNAs may compensate for the loss of the *miR-465* cluster in the male extraembryonic tissues, and that the *miR-465* cluster plays a sexual dimorphic role during extraembryonic tissues development. Comparisons between the *miR-465* KO male and WT male or between the KO non-degenerating female and WT female extraembryonic tissues at E7.5 found no upregulated miRNAs, whereas 74 miRNAs were found dysregulated in between the KO degenerating and KO non-degenerating females (**Figures S4D, E**; **Tables S5C–S5E**), suggesting that the degenerating females are more “sensitive” to the *miR-465* KO. Of interest, 26 miRNAs of the 74 dysregulated miRNAs all target *Alkbh1*, one of the validified targets of the *miR-465* cluster that has a sex dimorphic role during extraembryonic development (27). No significant changes in mRNA transcriptome were detected between either WT and KO males, or between WT and the KO non-degenerating females; however, drastic changes were observed between the KO degenerating and KO non-degenerating females. Among these dysregulated genes, some of them are either sex-specific (e.g., *Alkbh1* and *Rlim*) or imprinted genes, further confirming that the *miR-465* cluster influences the extraembryonic development in a sex-specific manner through mediating sex differential genes.

Spontaneous embryonic resorption during early pregnancy is common in most mammalian species, including mice, rats, rabbits, voles, ewes, red pandas, swine, and humans (35–44). Moreover, spontaneous embryonic resorption during early pregnancy does not necessarily lead to reduced litter size (35, 36). Given that the embryonic resorption occurs randomly without obvious sex ratio bias, it is highly likely that both male and female embryos are resorbed at a similar rate to maintain a balanced sex ratio. Some studies have correlated embryonic loss with aberrant placental development (44). A recent in-depth survey of 103 knockout mice lines that display embryonic lethality has revealed that ~68% of these embryonic resorption cases are caused by placental dysfunction (45). Our data that almost all of the resorbed *miR-465* KO embryos are females suggest a sexual dimorphic role of the *miR-465* cluster in extraembryonic/placental development. Like the other X-linked miRNA clusters (14), the *miR-465* has its orthologue in humans, which was named *miR-892b*, suggesting that the findings in mice may apply to humans. Supporting this hypothesis, a recent study in humans showed that the *miR-892b* was downregulated in the plasma collected from preeclampsia pregnancies (46), which is often accompanied by fetal growth restriction and placental abruption (47).

Taken together, our study uncovered an essential role of the *miR-465* cluster in supporting the full developmental potential of the female, but not the male, extraembryonic tissues/placentae (**Figure 4D**). The male-biased sex ratio among *miR-465* KO mice results from selective degeneration of the female placenta and resorption of the female embryos in the absence of the *miR-465* cluster.

## Data Availability Statement

The original contributions presented in the study are publicly available. This data can be found here: https://www.ncbi.nlm.nih.gov/bioproject/PRJNA669325/.

## Ethics Statement

The animal study was reviewed and approved by Institutional Animal Care and Use Committee (IACUC) of the University of Nevada, Reno (Protocol# 00494).

## Author Contributions

ZW and WY designed the research. Z W, NM, YW, SW, SC, and HZ performed bench experiments. ZW, TZ, and ML performed bioinformatic analyses. SK and HW contributed reagents and protocols. ZW and WY wrote the manuscript. All authors contributed to the article and approved the submitted version.

## Funding

This work was supported by grants from the NIH (HD098593, HD0085506, HD099924 to WY) and the Templeton Foundation (PID: 61174 to WY).

## Conflict of Interest

The authors declare that the research was conducted in the absence of any commercial or financial relationships that could be construed as a potential conflict of interest.

## Publisher’s Note

All claims expressed in this article are solely those of the authors and do not necessarily represent those of their affiliated organizations, or those of the publisher, the editors and the reviewers. Any product that may be evaluated in this article, or claim that may be made by its manufacturer, is not guaranteed or endorsed by the publisher.
