# Supplementary material for: Ablation of the miR-465 Cluster Causes a Skewed Sex Ratio in Mice
Source: Front Endocrinol (Lausanne). 2022 May 23;13:893854. doi: 10.3389/fendo.2022.893854 (PMC9167928; doi:10.3389/fendo.2022.893854)
Supplement: Supplementary file 1 [file DataSheet_1.zip › Supplementary Figures S1-S4.DOCX]

Supplementary Material - Figures

**
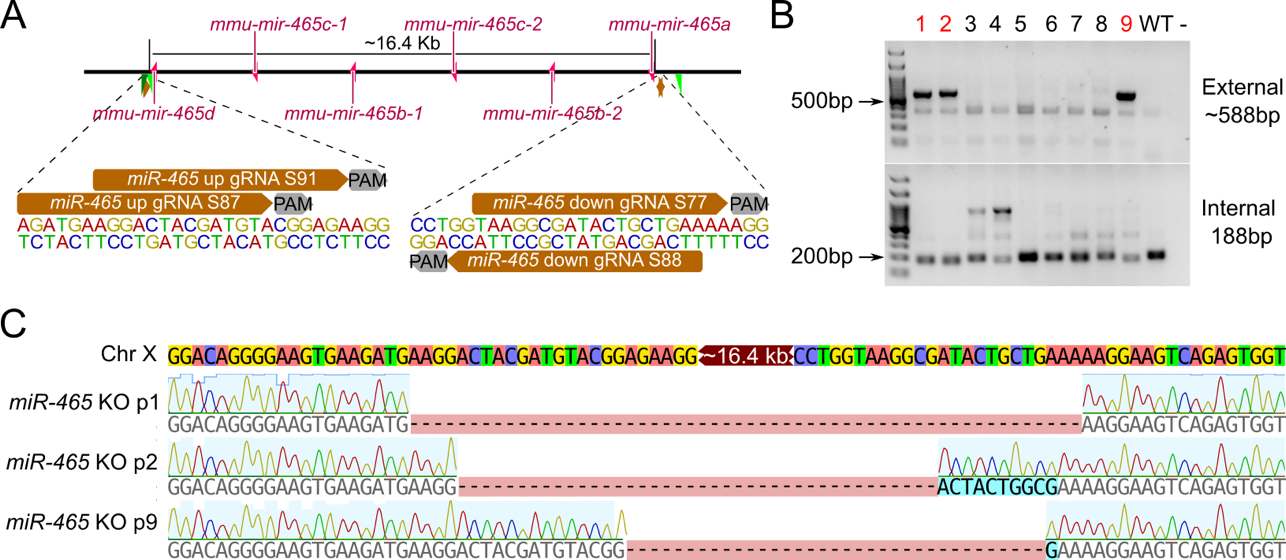
**

**Figure S1.** Generation of *miR-465* KO mice. A. Schematic illustration showing the strategy used to generate the *miR-465* KO mice using CRISPR-Cas9. The brown triangle represents the gRNA used, and its right and left orientations indicate the reverse and forward strands targeted by the gRNAs, respectively. The green triangles show the position of primers used for genotyping. B. A representative genotyping result of the *miR-465* KO founder mice. WT, wild-type. The expected size of PCR products is indicated on the right of the gel, the animal numbers in red represent the positive founders. C. Sanger sequencing results of the PCR bands from the three positive founder mice.


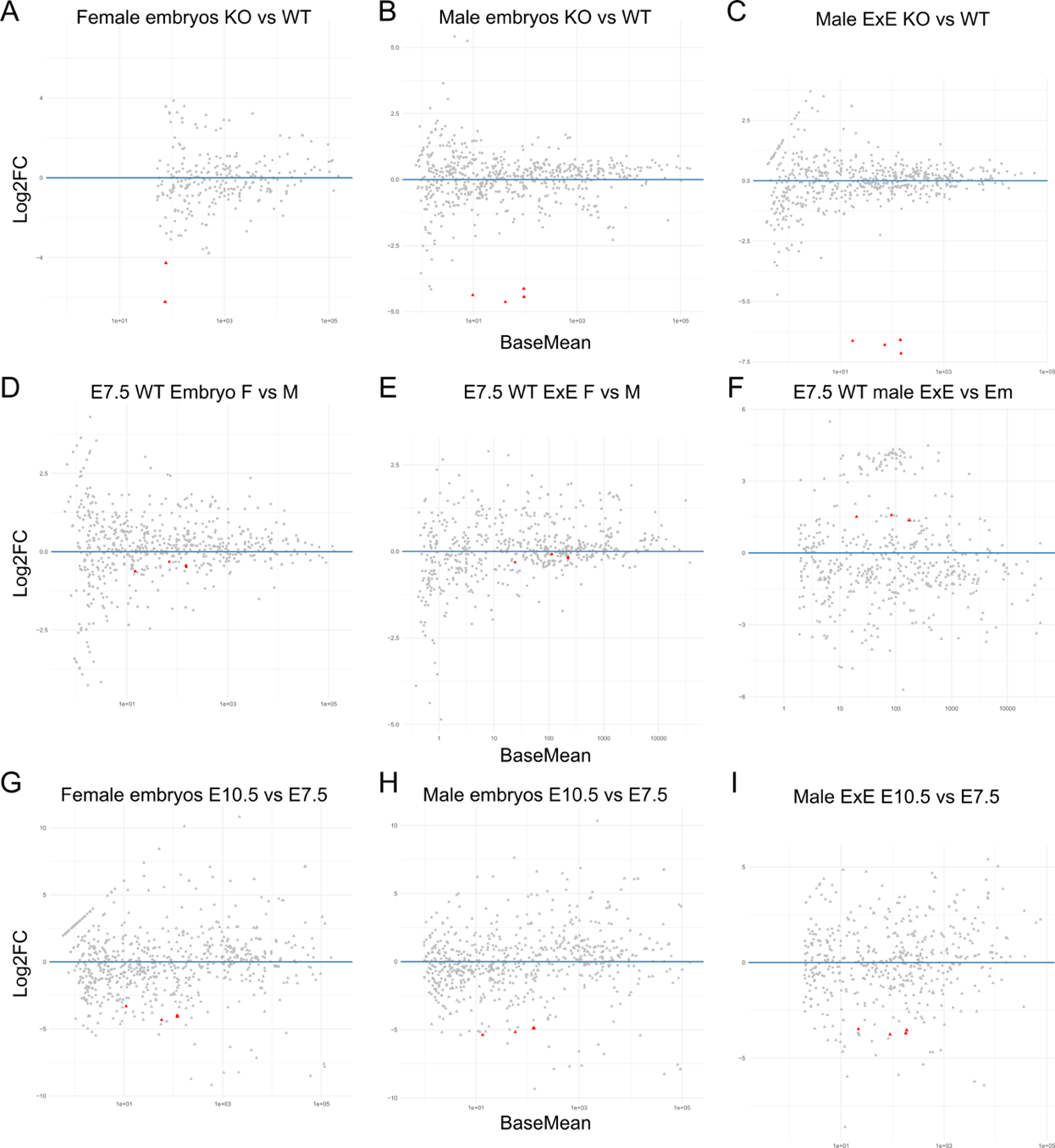


**Figure S2.** sRNA-seq analyses of the murine embryos and extraembryonic tissues/placenta at E7.5 and E10.5. A. Differentially expressed miRNAs between WT and KO female embryos at E7.5. B. Differentially expressed miRNAs between WT and KO male embryos at E7.5. C. Differentially expressed miRNAs between WT and KO male extraembryonic tissues at E7.5. D. Differentially expressed miRNAs between female and male embryos at E7.5. E. Differentially expressed miRNAs between female and male extraembryonic tissues in wild-type mice at E7.5. F. Differentially expressed miRNAs between extraembryonic tissues and embryos in wild-type males at E7.5. G. Differentially expressed miRNAs between E10.5 and E7.5 female embryos. H. Differentially expressed miRNAs between E10.5 and E7.5 male embryos. I. Differentially expressed miRNAs between E10.5 and E7.5 male extraembryonic tissues. sRNA-seq analyses were conducted in biological triplicates (n=3), ExE, extraembryonic tissues. Triangle and circle indicate FDR < 0.05 and FDR > 0.05, respectively. Dots in red indicate the *miR-465* cluster miRNAs.


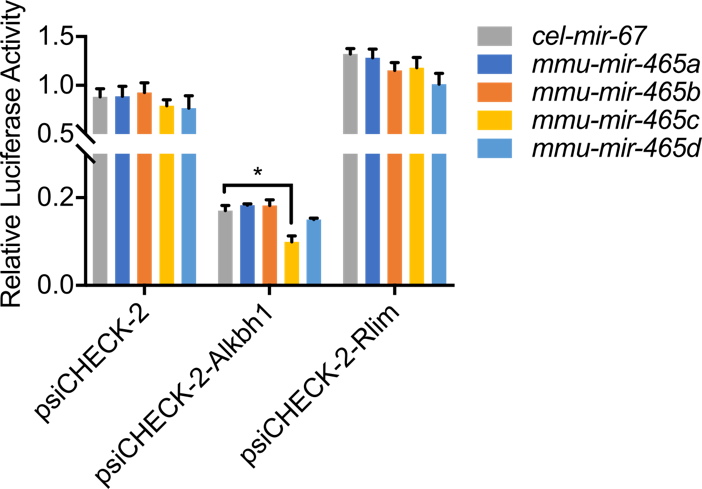


**Figure S3.** Luciferase reporter assays for *Alkbh1* and *Rlim*. psiCHECK-2 was the empty vector used; the *cel-mir-67* served as a negative control, and firefly luciferase activity was used as the internal control. *, p<0.05.

**
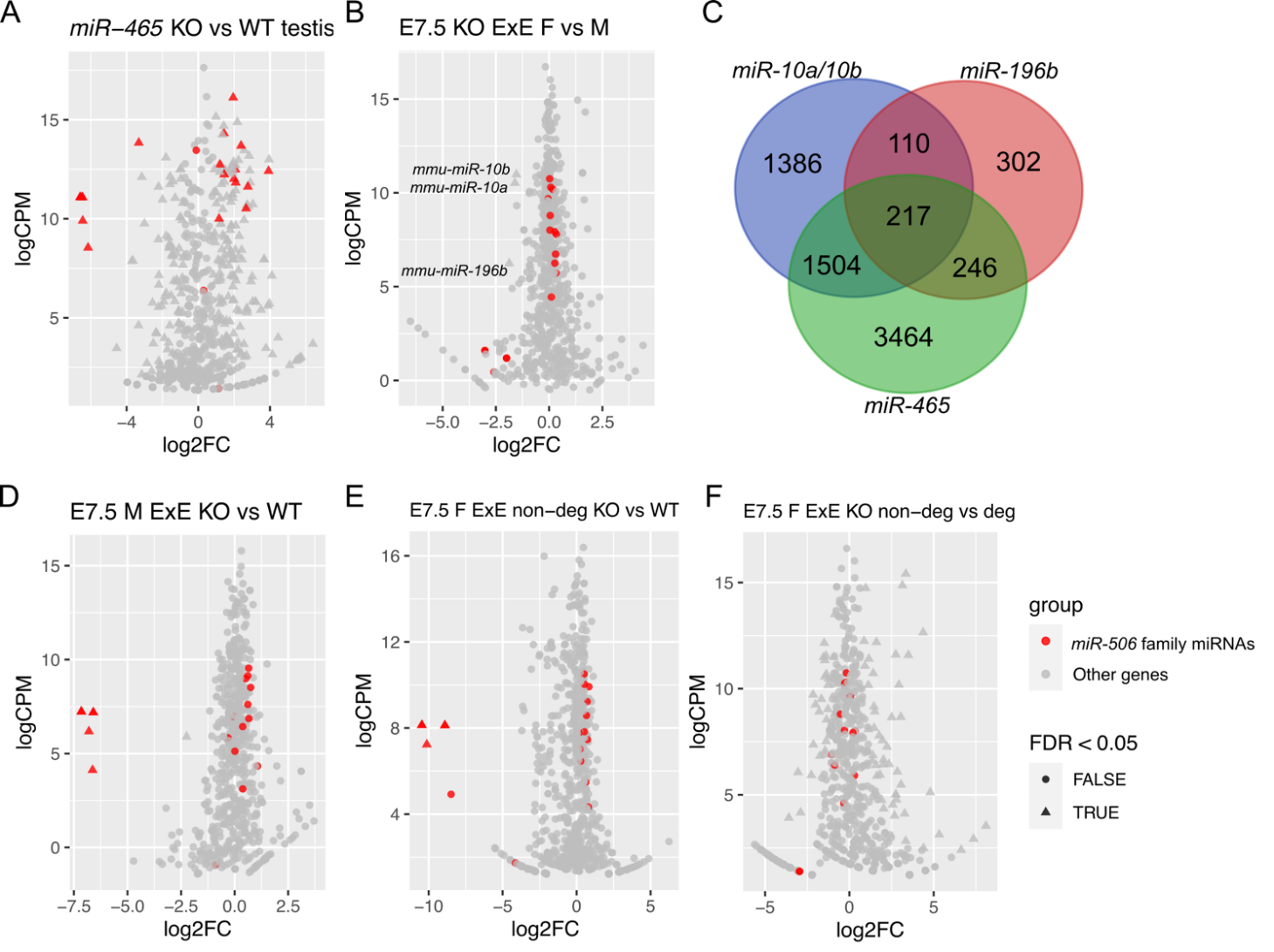
**

**Figure S4. Genetic compensation analysis for the *miR-465* cluster KO mice.** A. Differentially expressed miRNAs in the *miR-465* cluster KO and WT mice testes. B. Differentially expressed miRNAs in the *miR-465* cluster KO female and male extraembryonic tissues at E7.5. C. A Venn diagram showing the overlapped target genes among *miR-465*, *miR-10a/10b*, and *miR-196b*. D. Differentially expressed miRNAs in the *miR-465* cluster KO and WT male extraembryonic tissues at E7.5. E. Differentially expressed miRNAs in the *miR-465* cluster KO non-degenerating female and WT female extraembryonic tissues at E7.5. F. Differentially expressed miRNAs in the *miR-465* cluster KO non-degenerating and degenerating female extraembryonic tissues at E7.5. sRNA-seq analyses were conducted in biological triplicates (n=3), ExE, extraembryonic tissues. Triangle and circle indicate FDR < 0.05 and FDR > 0.05, respectively. Dots in red indicate the *miR-506* family miRNAs.
