# Supplementary material for: Ablation of the miR-465 Cluster Causes a Skewed Sex Ratio in Mice
Source: Front Endocrinol (Lausanne). 2022 May 23;13:893854. doi: 10.3389/fendo.2022.893854 (PMC9167928; doi:10.3389/fendo.2022.893854)
Supplement: Supplementary file 1 [file DataSheet_1.zip › Supplementary Tables S1 and S2.DOCX]

Supplementary Material – Tables S1 and S2

| **Table S1. Oligos used in this study.** | |  |
| --- | --- | --- |
| Name | Sequence | Usage |
| miR465 up gRNA1 S91 | AGGACTACGATGTACGGAGA | gRNA |
| miR465 up gRNA2 S87 | AGATGAAGGACTACGATGTA | gRNA |
| miR465 down gRNA1 S88 | TTCAGCAGTATCGCCTTACC | gRNA |
| miR465 down gRNA2 S77 | TAAGGCGATACTGCTGAAAA | gRNA |
| miR465 Ext F | GGCCTGATCTATTCTGAAGGGA | genotyping |
| miR465 Ext R | ATCTCACAAATGCCTTTCCGA | genotyping |
| miR465 Int R | GATGAGCTTGCACATATCCACA | genotyping |
| DyzEms3 F | TAGGATGGTAAGCCCAATGC | Sex determination |
| DyzEms3 R | TTGGTTGGTTAATTGTTTGGG | Sex determination |
| Rn18s F | AGAAACGGCTACCACATCCAA | Sex determination |
| Rn18s R | CCTGTATTGTTATTTTTCGTCACTACCT | Sex determination |
| mmu-miR-465a-5p | DIG-ucacaucagugccauucuaaaua-DIG | miRNA-ISH |
| mmu-miR-465a/b/c-3p | DIG-ucuacuuagaaaggcccugauc-DIG | miRNA-ISH |
| mmu-miR-465d-3p | DIG-uuacucagaaaggcccugauca-DIG | miRNA-ISH |
| mmu-miR-NC | DIG-uucuccgaacgugucacgutt-DIG | miRNA-ISH |
| LD_CDS | CAAGCAGAAGACGGCATACGAGATCGTGATGTGACTGGAGTTCAGACGTGTGCTCTTCCGATCTTTTTTTTTTTTTTTTTTTTTTVN | miRNA cDNA RT primer |
| Illu lib quant probe | /56-FAM/AGCACACGT/ZEN/CTGAACTCCAGTCAC/3IABkFQ/ | qPCR probe |
| Truseq anti | AGCAGAAGACGGCATACGA | qPCR reverse primer |
| miR-465a-5p | TATTTAGAATGGCACTGATGTGA | qPCR forward primer |
| miR-465b-5p | TATTTAGAATGGTGCTGATCTG | qPCR forward primer |
| miR-465c-5p | TATTTAGAATGGCGCTGATCTG | qPCR forward primer |
| miR-465-3p | GATCAGGGCCTTTCTAAGTAGA | qPCR forward primer |
| miR-465d-5p | TATTTAGAATGGTACTGATGTGA | qPCR forward primer |
| miR-465d-3p | TGATCAGGGCCTTTCTGAGTAA | qPCR forward primer |
| U6 qPCR F | ATACAGAGAAGATTAGCATGGCCC | qPCR forward primer |
| XhoI Rlim 3'UTR F | CGATCGCTCGAGTGAGACCAGAACTCTGAGCTGTGTA | Luciferase construct |
| NotI Rlim 3'UTR R | CGGCCAGCGGCCGCGATCACTTCATGCATTTTATTGATAGTCTATATTTTAAGTCTGC | Luciferase construct |
| XhoI Alkbh1 3'UTR F | CGATCGCTCGAGAACTTAGGGGCTAGATTCTCTTACTCAGTCA | Luciferase construct |
| NotI Alkbh1 3'UTR R | CGGCCAGCGGCCGCTTTAATTTAAAATAGAGTAACTTTATTCATGGAGAAAACAGCTAA | Luciferase construct |

**Table S2. Sex ratio bias in the literature**

| **Sex ratio (male %)** | **Condition** | **Species** | **References (PMID)** |
| --- | --- | --- | --- |
| 60% | *in vitro* fertilization | Mice | 26951653 |
| 67% | Very high in saturated fat | Mice | 12672968 |
| 39% | Low in saturated fat | Mice | 12672968 |
| 58%~76% | *Mcm2/4* mutation | Mice | 30787433 |
| 52%~59% | *Alkbh1* mutation | Mice | 21072209 |
| 60% | Excess fructose consumption | Rats | 23759309 |
